# Case Report: Three cases of drug-coated balloon-only strategy for coronary artery lesions with aneurysms in the chronic phase

**DOI:** 10.3389/fcvm.2025.1710371

**Published:** 2025-11-12

**Authors:** Yu Sugawara

**Affiliations:** Cardiovascular Medicine, Yamato Kashihara Hospital, Kashihara-shi, Nara-ken, Japan

**Keywords:** coronary aneurysm, percutaneous coronary intervention, drug-coated balloon, cardiac computed tomography, case series, prognosis

## Abstract

**Introduction:**

Coronary aneurysms are rare; therefore, treatment strategies for coronary lesions with aneurysms remain elusive. We describe three cases of coronary artery lesions with aneurysms in patients treated with drug-coated balloons (DCBs).

**Case description:**

Case 1 had ST-segment elevation myocardial infarction with a right coronary artery aneurysm treated with a paclitaxel DCB. Case 2 had stenoses of the left anterior descending artery and diagonal branch, along with a coronary aneurysm treated using paclitaxel DCBs. Case 3 had a calcified left anterior descending artery with an aneurysm treated using a paclitaxel DCB. The acute outcomes of these cases were favorable, and prognoses based on follow-up computed tomography angiography findings in the chronic phase were also favorable.

**Conclusion:**

DCBs may be one option for treating coronary artery stenosis or occlusion.

## 1 Introduction

The incidence of coronary aneurysms is rare, at 0.3%–5.3% (1); hence, there are no effective treatment strategies for coronary lesions with aneurysms. Drug-eluting stent (DES) placement is the first choice of treatment for coronary stenoses or occlusions. However, coronary lesions with aneurysms are not suitable for DES placement, because enlarged coronary arteries hamper adequate stent deployment. Additionally, stent malapposition can cause stent thrombus (2). Drug-coated balloons (DCBs) are novel devices that enable a stentless treatment strategy, potentially shortening the duration of dual antiplatelet therapy (DAPT) (3). Furthermore, the indications for DCBs are expanding; they are now used in large vessels and acute coronary syndrome (4, 5). Here, we describe the outcomes of three cases of coronary artery lesions with aneurysms treated with DCBs.

## 2 Case description and diagnostic assessment

### 2.1 Ethics statement

This manuscript is original, has not been published previously, and is not under consideration for publication elsewhere. This study received ethical approval from the institutional review board of our hospital, and written informed consent was obtained from all participants.

### 2.2 Case 1

**Figure 1:**
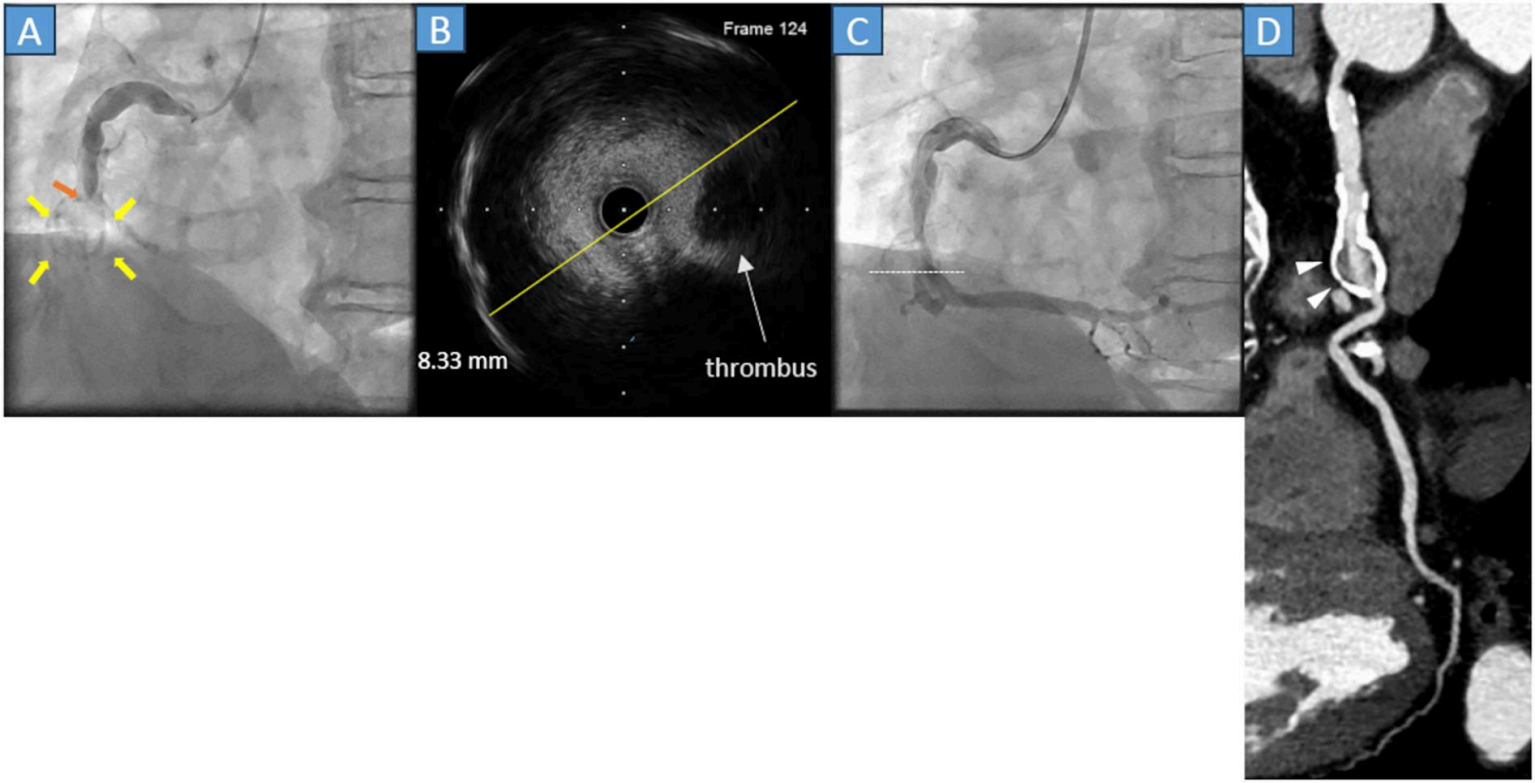
**(A)** Coronary angiography (CAG) reveals acute total occlusion of the middle right coronary artery (orange arrow) with a circular calcified structure (yellow arrows). **(B)** The cross-sectional image of the intravascular ultrasound (IVUS) reveals that the diameter of the lesion was 8.33 mm (yellow line) with thrombus and calcification of the vessel wall. **(C)** The final CAG reveals a Thrombolysis in Myocardial Infarction grade III flow. The white dots in the angiography correspond to the cross-sectional IVUS image. **(D)** Cardiac computed tomography in the chronic phase reveals that preserved right coronary flow and the presence of a saccular aneurysm with severe calcification (gray arrowheads).

A 60-year-old man with hypercholesterolemia visited our emergency department because his symptoms had worsened 6 h before hospital admission. His history of Kawasaki disease was unclear. Electrocardiography (ECG) showed ST-segment elevation in leads II, III, and aVF, and his cardiac troponin T level was 0.030 ng/mL (normal range, <0.014 ng/mL). As an acute inferior ST-elevation myocardial infarction (STEMI) was suspected, urgent coronary angiography (CAG) was performed, and revealed total occlusion of the middle segment of the right coronary artery (RCA) (Figure 1A, orange arrow), along with severe calcification of the aneurysm (Figure 1A, yellow arrows). In March 2024, subsequently, percutaneous coronary intervention (PCI) was performed. A 0.014-inch guidewire was introduced, then intravascular ultrasound (IVUS) was employed. IVUS revealed an exceeding 8 mm coronary aneurysm (Figure 1B yellow line, Figure 1C white dots), thrombus, severe calcification, and substantial differences in diameter between the distal and proximal lesions. Stent placement was avoided, owing to IVUS findings. A 3.0*20 mm paclitaxel DCB angioplasty was performed following predilatation with a 3.0*13 mm scoring balloon. The final CAG revealed thrombolysis in myocardial infarction grade III flow in the RCA (Figure 1C) without dissection. After PCI, the patient was prescribed aspirin, clopidogrel, and statins for five months. After five months, clopidogrel was discontinued. Aspirin, warfarin, and statins were administered. During follow-up, he reported no chest symptoms.

Approximately 1 year and 1 month after PCI (April 2025), cardiac coronary computed tomography (CT) was performed and showed the lesion remained patent with a saccular right coronary artery aneurysm with heavy calcification but no enlargement compared with the lesion size in the acute phase (Figure 1D, white arrowheads).

### 2.3 Case 2

**Figure 2:**
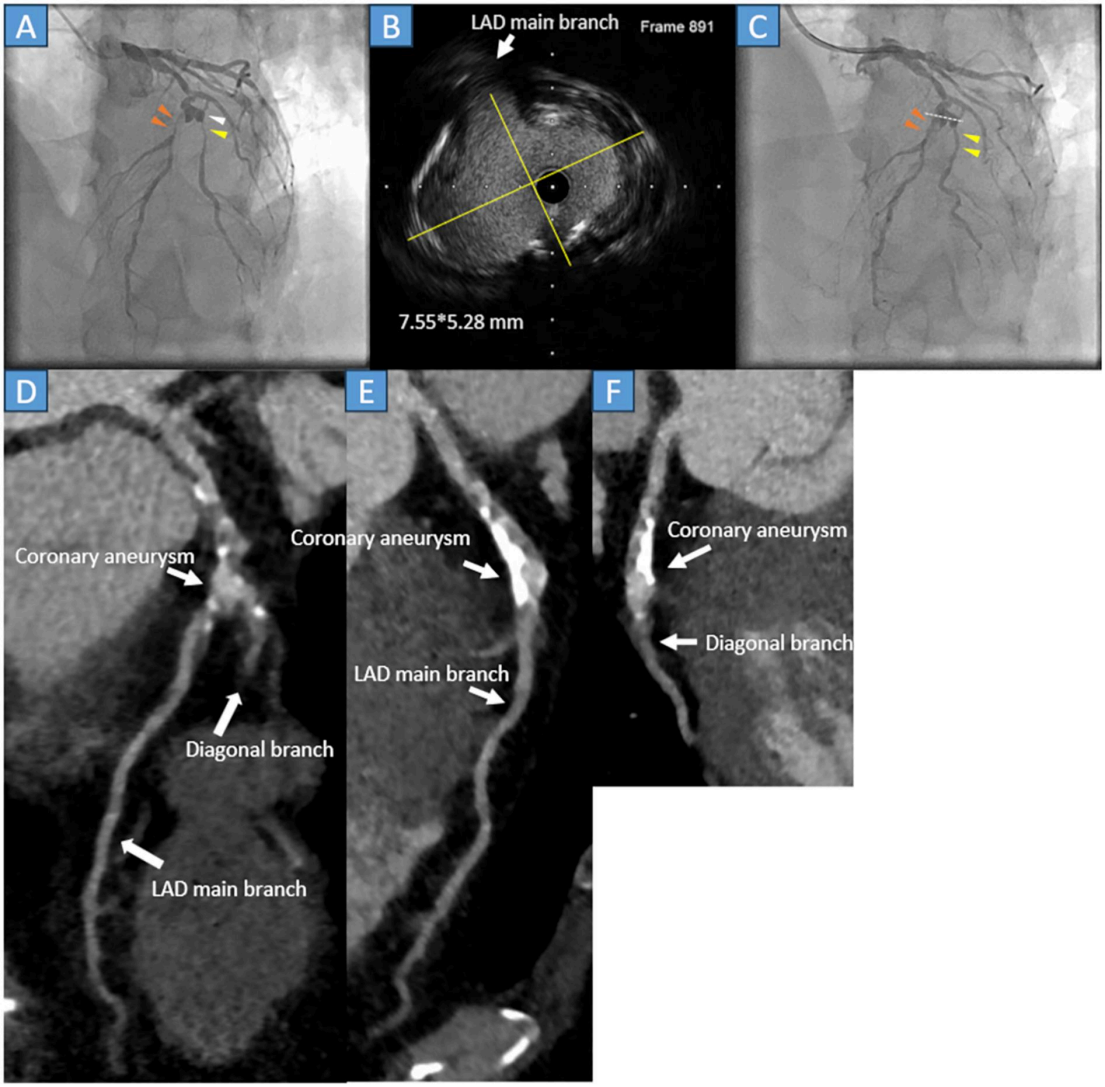
**(A)** Coronary angiography reveals severe stenosis at the middle segment of the left anterior descending artery (LAD) (orange arrowheads) and the second diagonal branch (yellow arrowhead), just after the aneurysm formation (white arrowhead). **(B)** The cross-sectional image of the intravascular ultrasound (IVUS) detects a 7.55*5.28 mm (yellow lines) mild calcified oval-shaped coronary aneurysm. **(C)** The LAD (orange arrowheads) and second diagonal branch (yellow arrowheads) were successfully treated with the drug-coated balloon. The white dots in the angiography correspond to the cross-sectional IVUS image. **(D)** Cardiac computed tomography showing patient LAD and secondary diagonal branches with calcification. **(E)** The LAD is patent with atherosclerotic change and calcification. **(F)** The second diagonal branch is patent, and the main branch of the LAD is severely calcified.

A 54-year-old male with an unknown medical history, but no prior Kawasaki disease, was admitted to our hospital because of effort angina. The treadmill stress test result was positive; therefore, an elective CAG was performed. The CAG revealed stenoses and an aneurysm in the left anterior descending artery (LAD) (Figure 2A, orange arrowheads) and diagonal branch (Figure 2A, yellow arrowheads). Subsequently, in April 2022, PCI was performed. After crossing the 0.014-inch guidewire, IVUS was performed. IVUS revealed a mildly calcified lesion and a 7.55*5.28 mm oval-shaped aneurysm (Figure 2B yellow lines, Figure 2C white dots). The LAD was treated with a 2.5*20 mm paclitaxel DCB dilating a 2.0*13 mm scoring balloon, and the diagonal branch was treated with a 2.0*15 mm paclitaxel DCB after dilating a 2.0*15 mm compliant balloon.

The immediate outcome was favorable (Figure 2C, orange and yellow arrowheads) without dissection. Post-procedure, the patient was prescribed three months of aspirin, prasugrel, statins, and beta blockers; after three months, prasugrel was discontinued. During the follow-up, he did not report any chest discomfort. Approximately 3 years and 3 months later (July 2025), a repeat cardiac CT was performed. The scan revealed moderate atherosclerotic changes in the LAD. However, no stenosis was observed in either the LAD or diagonal branch (Figures 2D–F, white arrows).

### 2.4 Case 3

**Figure 3:**
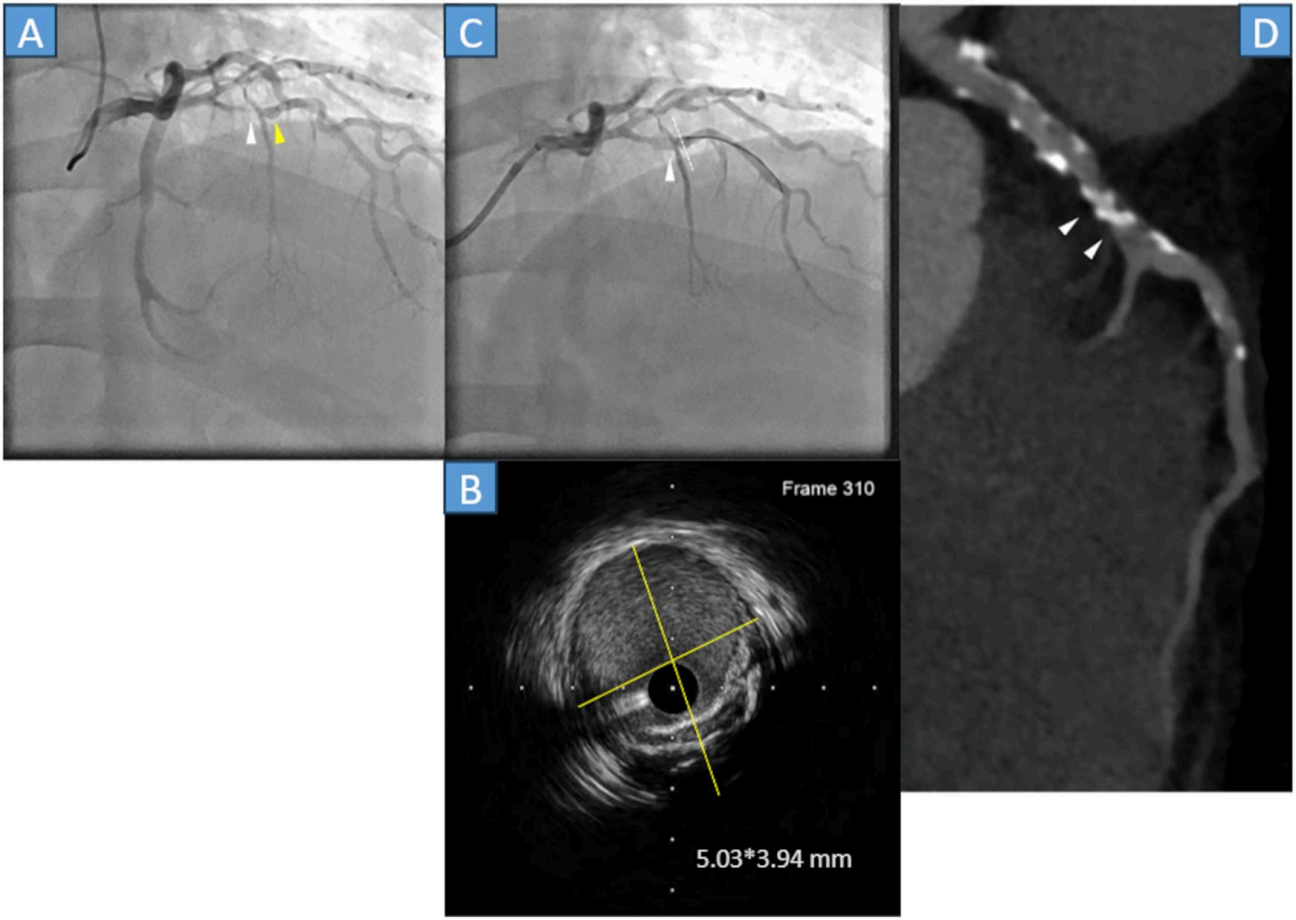
**(A)** Coronary angiography (CAG) showed stenosis just before the septal branch (white arrowhead) and aneurysm (yellow arrowhead). **(B)** The cross-sectional image of the intravascular ultrasound (IVUS) indicates a 5.03*3.94 mm aneurysm with calcification (yellow lines). **(C)** Final CAG reveals that the left anterior descending (LAD) artery was dilated with National Heart, Lung, and Blood Institute grade A dissection (white arrowhead). The white dots in the angiography correspond to the cross-sectional IVUS image. **(D)** Cardiac computed tomography indicates that the calcified lesion was patent (white arrowheads).

A 49-year-old male with a history of stent-assisted coiling of an intracranial aneurysm, hypertension, and dyslipidemia without Kawasaki disease was admitted to our hospital because of chest pain. The ECG did not show any substantial changes; however, the cardiac troponin T level was 0.083 ng/mL (normal range, <0.014 ng/mL). Emergency CAG was performed because of a non-STEMI. The CAG demonstrated that the proximal LAD artery had severe stenosis (Figure 3A, white arrowhead) with an aneurysm (Figure 3A, yellow arrowhead). Thus, in April 2022, PCI was performed. A 0.014-inch guidewire was crossed, and IVUS was performed. IVUS revealed a 5.03*3.94 mm aneurysm and lesion calcification (Figure 3B yellow lines, Figure 3C white dots). After dilating the lesion with a 2.5*13 mm scoring balloon, a 3.0*15 mm paclitaxel DCB was used; final CAG showed a fusiform coronary aneurysm, whose flow with National Heart, Lung, and Blood Institute grade A dissection (Figure 3C, white arrowhead). Following PCI, the patient was asymptomatic, and medication therapy: Aspirin, clopidogrel, statin, and ezetimibe. After 3 months, clopidogrel was discontinued. Approximately 3 years and 3 months later (July 2025), cardiac CT was performed. The LAD was calcified, and the target lesion was patent (Figure 3D, white arrowheads).

## 3 Discussion

We experienced the three cases of coronary occlusion or stenosis with aneurysms successfully treated with a paclitaxel DCB. Follow-up was performed during the chronic period, and imaging was performed to assess favorable outcomes. In these cases, the etiology of the aneurysm was unclear; however, it may have been related to Kawasaki or atherosclerotic disease.

The rationale for the DCB-only strategy is that, generally, a DCB would have no triggers for thrombosis, such as uncovered struts and metal allergy, unlike stents. The absence of foreign materials reduces the risk of target lesion thrombosis. Additionally, the DCB-only strategy can shorten the duration of DAPT, potentially reducing bleeding events and making surgery easier for the patient (3). Furthermore, if a target lesion failure, like thrombosis or restenosis occurs, multiple strategies are considered compared with stent implantation because the DCB-only strategy does not involve metallic cages.

Several coronary interventional strategies are available to treat coronary ischemia with aneurysms, including stent implantation (e.g., covered stent implantation), conventional balloon angioplasty [plain old balloon angioplasty (POBA)], excimer laser coronary angioplasty (ELCA), and DCB therapy. Stent implantation is considered the first option. However, because the presented patients have coronary aneurysms, stent malapposition, in-stent restenosis, and stent thrombus could have occurred. Thus, a covered stent was considered (6); however, in our cases, a high incidence of in-stent restenosis after using the covered stent in the chronic phase was a concern. Furthermore, the coronary anatomy is not suitable for covered stent implantation because of the presence of side branches. Therefore, stent therapy was not adopted. Consequently, conventional POBA was considered; however, it is associated with a high probability of restenosis during the chronic phase. Although ELCA seems promising, few hospitals in our country can use it. Finally, DCB therapy has potential advantages, as it is simple to implement. Case 1 had STEMI, case 2 had bifurcation lesions, and case 3 had non-STEMI, and there have been studies on the effectiveness of DCB concerning STEMI (5), bifurcation lesions (7), and non-STEMI (8). As aforementioned, we opted for a DCB-only strategy.

There is a report that ST-segment elevation myocardial infarction with right coronary aneurysm was successfully treated with a drug-coated balloon (9). However, long term follow-up data on coronary ischemia in patients with aneurysms after PCI are limited. The reported data show that the incidence of major adverse cardiac events was higher in patients with coronary artery aneurysms than in those compared with without coronary aneurysm after DES implantation (10). In addition, regarding outcomes of PCI for STEMI, the coronary artery aneurysm group had early stent thrombosis due to recurrent myocardial infarctions compared with its counterpart (11). Furthermore, the outcomes of DCB therapy for coronary ischemia with a coronary aneurysm are limited. There have been some case reports of coronary artery ischemia treated with DCB, with favorable outcomes (12, 13). However, there are still insufficient supportive data.

After PCI, medical therapy was considered for patients who had coronary artery stenosis with aneurysm. Generally, patients treated with paclitaxel DCB are recommended to receive DAPT for one to three months. After completing DAPT, single antiplatelet therapy is continued (14). Anticoagulation therapy is recommended for acute coronary syndrome with a giant aneurysm (diameter ≥ 8 mm) because aspirin and warfarin combination therapy reduces the incidence of myocardial infarction (15, 16). The long-term prognosis of a coronary artery aneurysm is unfavorable because concomitant atherosclerotic factors can lead to cardiac events (17); therefore, aggressive risk factor modification of risk factors is necessary. An overview of medical therapy during the chronic phase; Case 1: The patient, having experienced STEMI accompanied by a giant aneurysm, was prescribed aspirin, warfarin, and a statin. Case 2: The therapeutic regimen included aspirin, a statin, and a beta blocker. Case 3: The patient received aspirin, a statin, and ezetimibe. Antiplatelet agents, statins, ezetimibe, and beta blockers were administered as part of secondary prevention strategies. Anticoagulation therapy was administered in Case 1 due to the presence of STEMI in conjunction with a giant coronary artery aneurysm, defined by a diameter ≥ 8 mm. The substantial thrombotic risk associated with giant coronary artery aneurysms, compounded by the acute ischemic presentation, warranted the initiation of systemic anticoagulation to mitigate the potential for further coronary occlusion. In contrast, Cases 2 and 3 involved medium-sized coronary artery aneurysms (diameter between 4 and 8 mm), for which warfarin was not indicated (18, 19). Consequently, a strategy of short-term DAPT was employed during the acute phase, followed by maintenance with aspirin monotherapy to balance thrombotic protection with bleeding risk.

Coronary ischemia associated with a coronary aneurysm is often complex and challenging. Thus, a DCB-only strategy may be the key to obtaining favorable outcomes in such cases.

## 4 Limitations

This case series has some limitations.

First, although the DCB-only strategy was favorable, only three cases patients were evaluated. More cases and studies are needed to strengthen our conclusion.

Second, only SeQuent Please paclitaxel DCBs were used. Use of other DCBs may affect the results.

Third, these patients were evaluated using CT angiography, and did not undergo follow-up CAG, so coronary stenoses were not assessed accurately.

Finally, as the follow-up duration was approximately 1–3 years, longer follow-up periods are necessary to corroborate the efficacy of the DCB-only strategy.

## 5 Conclusions

A DCB-only strategy for treating coronary artery disease patients with aneurysms is effective and has favorable outcomes. DCBs are one of the options for treating coronary stenoses with aneurysms.

## 6 Patient perspective

Patients who had coronary ischemia with coronary aneurysms treated with DCBs had no foreign materials in their bodies. Thus, this strategy is a patient-friendly method.

## Data Availability

The datasets presented in this study can be found in online repositories. The names of the repository/repositories and accession number(s) can be found in the article/Supplementary Material.
